# T7 replisome directly overcomes DNA damage

**DOI:** 10.1038/ncomms10260

**Published:** 2015-12-17

**Authors:** Bo Sun, Manjula Pandey, James T. Inman, Yi Yang, Mikhail Kashlev, Smita S. Patel, Michelle D. Wang

**Affiliations:** 1 Department of Physics, Laboratory of Atomic and Solid State Physics, Cornell University, Ithaca, New York 14853, USA; 2 Howard Hughes Medical Institute, Cornell University, Ithaca, New York 14853, USA; 3 School of Life Science and Technology, ShanghaiTech University, Shanghai 201210, China; 4 Department of Biochemistry and Molecular Biology, Rutgers-Robert Wood Johnson Medical School, Piscataway, New Jersey 08854, USA; 5 NCI Center for Cancer Research, Frederick, Maryland 21702, USA

## Abstract

Cells and viruses possess several known ‘restart' pathways to overcome lesions during DNA replication. However, these ‘bypass' pathways leave a gap in replicated DNA or require recruitment of accessory proteins, resulting in significant delays to fork movement or even cell division arrest. Using single-molecule and ensemble methods, we demonstrate that the bacteriophage T7 replisome is able to directly replicate through a leading-strand cyclobutane pyrimidine dimer (CPD) lesion. We show that when a replisome encounters the lesion, a substantial fraction of DNA polymerase (DNAP) and helicase stay together at the lesion, the replisome does not dissociate and the helicase does not move forward on its own. The DNAP is able to directly replicate through the lesion by working in conjunction with helicase through specific helicase–DNAP interactions. These observations suggest that the T7 replisome is fundamentally permissive of DNA lesions via pathways that do not require fork adjustment or replisome reassembly.

## Abstract (web summary)

Genomic instability can result from stalled or collapsed replication fork at sites of unrepaired DNA lesions. Here the authors uncover a new lesion bypass pathway for the T7 replisome, where leading strand template lesions can be overcome through interaction between the replisome's helicase and polymerase components.

## Graphical abstract

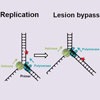

DNA lesions, if not repaired, may cause a replication fork to stall or collapse, leading to genomic instability and cell death^1^. To avoid replication failure, cells rely on DNA damage tolerance and DNA repair pathways to complete the replication cycle^2^,^3^. Lesions in the lagging strand are generally not major obstacles for replication fork progression; however, leading-strand DNA lesions present substantial barriers for fork progression^4^,^5^,^6^,^7^,^8^. Several pathways have been proposed to explain how a replication fork, stalled by a leading-strand block, can be restarted: (1) fork reversal, where the leading strand is extended via a template switch, which utilizes the nascent lagging strand as a template^9^,^10^; (2) lesion skipping, where leading-strand synthesis is reinitiated downstream from the lesion via a primase-dependent priming event, ultimately leaving a gap in the newly replicated DNA that will need to be filled later^11^; and (3) translesion synthesis, where the replicative DNA polymerase (DNAP) is transiently replaced by a translesion DNAP that allows for replication through a lesion because the active site of a translesion DNAP can accommodate various damaged template bases^12^. Although some of these pathways may occur in a rapid manner^13^, others may require the recruitment of multiple proteins and/or reassembly of the replisome before the re-initiation of replication. These requirements likely postpone replication fork movement and may even lead to cell cycle arrest^1^. This cost, coupled with evidence that lesions do not present a complete block to replication^14^, suggests the possibility of a more direct mechanism to overcome lesions.

To monitor the dynamic process of DNA replication in real time and determine the fates of each replicative protein after encountering a lesion, we developed a single-molecule assay, using the bacteriophage T7 replisome, to examine the effect of a *cis-syn* cyclobutane pyrimidine dimer (CPD) lesion in a leading-strand DNA template. We found that replicative T7 DNAP alone is incapable of lesion bypass. However, with the assistance of T7 helicase, T7 DNAP is able to directly overcome the CPD lesion. In this process, T7 helicase stays with the paused DNAP at the lesion and then they concurrently resume their activities after the lesion. Ensemble data further confirm these results. Furthermore, both single-molecule and ensemble data demonstrate that this lesion-overcoming behaviour is a result of a helicase-coupled wild-type (wt) DNAP directly synthesizing through the lesion and continuing replication. Our work is the first observation, to our knowledge, of CPD tolerance by a helicase-coupled wt replicative polymerase synthesizing through a lesion, rather than circumventing it. These results exhibit that the T7 replisome can tolerate and directly overcome leading-strand template lesions via interactions between helicase and polymerase, suggesting a new lesion bypass pathway.

## Results

### T7 helicase smoothly unwinds through a CPD lesion

**Figure 1:**
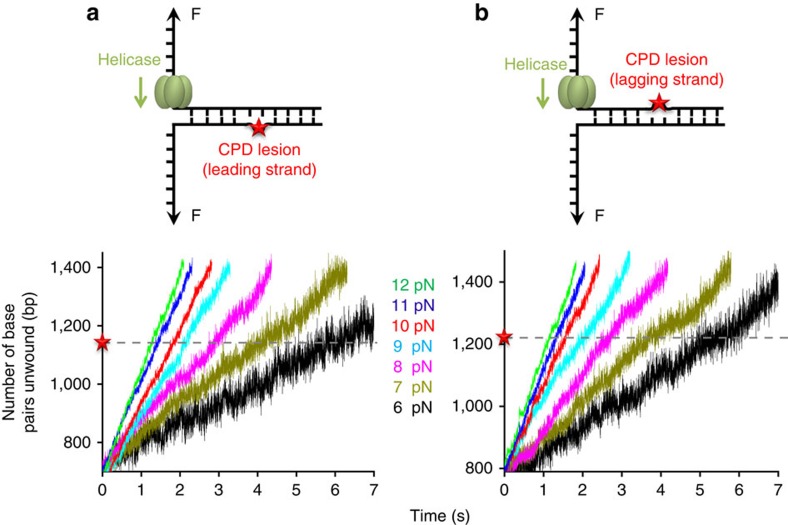
Helicase unwinding through a *cis-syn* CPD lesion. The two single-stranded ends of a dsDNA were held at a constant unzipping force of 6–12 pN as T7 helicase unwound the dsDNA. A CPD lesion (red star) was located in either the leading- (**a**) or lagging- (**b**) strand DNA. Representative traces show the number of unwound base pairs versus time in the presence of 2-mM Thymidine triphosphate (dTTP). For clarity, traces have been shifted along the time axis. The dotted lines indicate the lesion position.

In the T7 replisome, helicase paves the way for replication by separating double-stranded DNA (dsDNA) as it translocates along the lagging-strand DNA^15^,^16^. A previous study showed that translocation of T7 helicase on single-stranded DNA (ssDNA) was stalled by a bulky DNA adduct^17^; thus, we first measured T7 helicase unwinding of a CPD lesion-containing dsDNA. In our study, two strands of a DNA fork junction were held under a constant force that was not sufficient to mechanically unzip the junction, and helicase unwinding of the junction resulted in an increase in ssDNA length, permitting monitoring of helicase unwinding^18^,^19^ (Supplementary Fig. 1b). The CPD lesion was located on either the leading (Fig. 1a) or the lagging strand (Fig. 1b) of the dsDNA-unwinding substrate (Supplementary Fig. 1a). We found that, for both substrates, helicase smoothly unwound dsDNA without detectable stalls or pauses at the lesion position, with force-dependent unwinding rates indistinguishable from those for unmodified dsDNA (Fig. 1 and Supplementary Fig. 2). It is intuitive that the leading-strand CPD lesion would not impede T7 helicase unwinding, as the helicase translocates on the lagging stand. The smooth unwinding through the lagging-strand lesion suggests that the CPD lesion, unlike the bulky DNA adduct^17^, may fit in the central channel of T7 helicase, while the interactions between ssDNA and multiple helicase subunits^19^ may prevent helicase slippage or stalling.

### T7 DNAP alone is incapable of CPD lesion bypass

**Figure 2:**
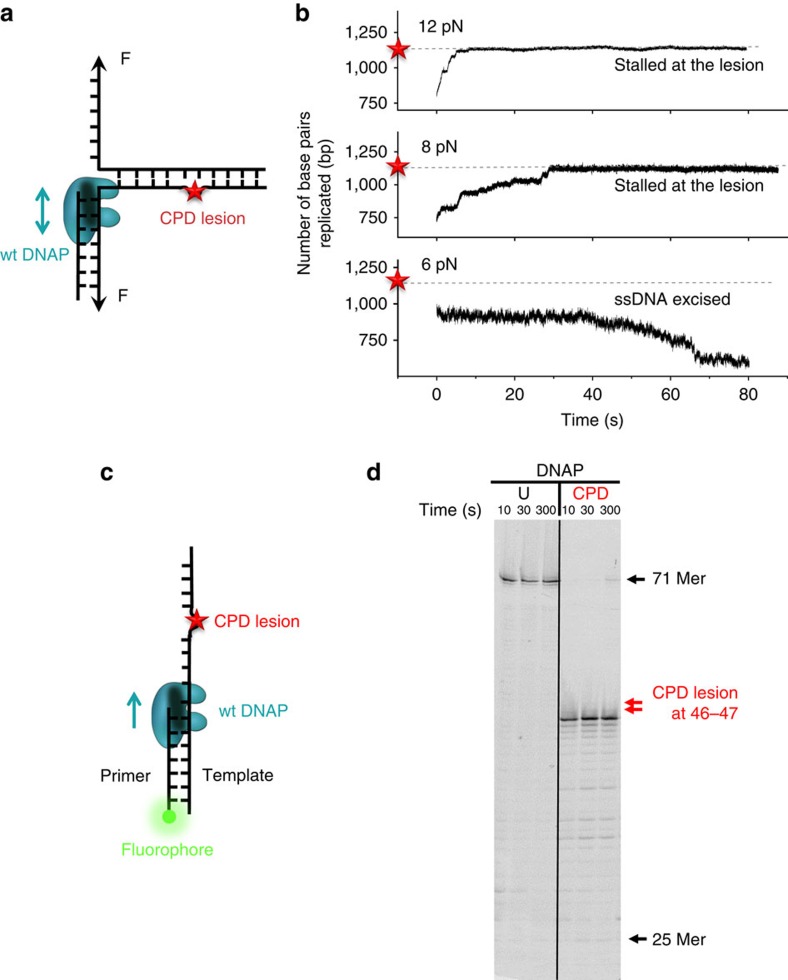
DNAP alone on a DNA template containing a CPD lesion. (**a**) Schematic representation of the single-molecule configuration. Two ssDNA arms were held at a constant force, while the motion of a T7 DNAP was monitored by the fork location. A single CPD lesion (red star) was located on the template strand. (**b**) Representative traces showing the number of replicated base pairs versus time in the presence of 1-mM dNTP (each) under 12, 8 and 6 pN. For clarity, traces have been shifted along the time axis. The dotted lines indicate the lesion position. Note that at 6 pN, DNAP excised DNA from the 3′ end. (**c**) Schematic representation of primer extension on a DNA template containing a single CPD lesion in ensemble studies. A 25-mer primer labelled with 5′ fluorescein was annealed to a 71-mer template containing a single CPD lesion at nucleotides 46 and 47. (**d**) A denaturing PAGE analysis of primer extension by DNAP on either an unmodified template (no CPD lesion, denoted as ‘U') or a CPD-containing DNA template (denoted as ‘CPD').

T7 DNAP is responsible for rapidly and faithfully copying genomic DNA in the T7 replisome^20^. It has a 3′ to 5′ exonuclease and proofreading activity to ensure the fidelity of DNA replication; however, *in vivo*, it lacks the ability to unwind dsDNA^21^. We investigated whether, in the absence of helicase, the replicative T7 DNAP is capable of directly replicating through a lesion. We first mechanically unzipped hundreds of base pairs of dsDNA to generate ssDNA and then allowed DNAP to perform the primer extension. After the DNAP encountered the fork, it began the strand displacement synthesis and we tracked its progress by monitoring DNA extension of the forked template as the two strands of a DNA fork were held under tension. Under a force of 8–15 pN that facilitated fork opening but was not sufficient to mechanically unzip the junction, DNAP displaced the other strand while synthesizing (Fig. 2a and Supplementary Fig. 1c)^22^. We used either a wt DNAP, which has both synthesis and exonuclease activities^20^, or an exonuclease-deficient (exo^−^) DNAP mutant, and found that both were able to replicate up to the lesion (Fig. 2b and Supplementary Fig. 3b). On encountering the lesion, wt DNAP was completely blocked, and no bypass was observed in any of the traces within 2 min (experimental cutoff time; Fig. 2b). In contrast, 65% of the exo^−^ DNAP mutants replicated through the lesion, with or without pausing, while the remaining exo^−^ DNAP mutants were stalled at the lesion (Supplementary Fig. 3b). These results indicate that T7 DNAP's exonuclease activity hinders its ability to overcome the CPD lesion. Under a low force (<8 pN), we observed a decrease in DNA extension with wt DNAP (Fig. 2b) and an unchanged extension with exo^−^ DNAP (Supplementary Fig. 3b), such that the lesion could not be reached in either case. The decrease in DNA extension with wt DNAP corresponds to a decrease in the number of base pairs replicated, most likely because of the exonuclease activity of wt DNAP under the influence of the regression pressure from the re-annealing fork^22^. At higher forces, this pressure is significantly alleviated and thus the processive exonuclease activity was not observed, even after wt DNAP pauses at the lesion for a long period of time (Fig. 2b).

To rule out that wt DNAP's inability to synthesize through the lesion was due to the presence of the DNA fork in the strand displacement configuration (Fig. 2a), we conducted a single-nucleotide resolution primer extension experiment using a 5′ fluorescein-labelled primer and a CPD lesion-containing ssDNA template with either wt or exo^−^ DNAP (Fig. 2c and Supplementary Fig. 3c). Control experiments, utilizing wt DNAP on an unmodified template, showed full extension (71 bp) within 10 s without pausing (Fig. 2d). In contrast, on a CPD-containing template, within 300 s, only 3% of the primers were fully extended and 43% of the primers stalled at position 45, just before the lesion at 46–47 positions (Fig. 2d). Exo^−^ T7 DNAP paused on the lesion template, but 29% reached full extension within 300 s (Supplementary Fig. 3d). These results are consistent with our single-molecule experiments on primer extension (Supplementary Fig. 4) as well as previous results^23^,^24^. The exonuclease activity of the DNAP provides a kinetic pathway to reverse DNA synthesis, and in the absence of this pathway (that is, exo^−^ DNAP), the forward DNA synthesis becomes the sole pathway. Taken together, we conclude that wt T7 DNAP is unable to independently synthesize past a CPD lesion.

### Helicase-coupled T7 DNAP synthesizes through a CPD lesion

**Figure 3:**
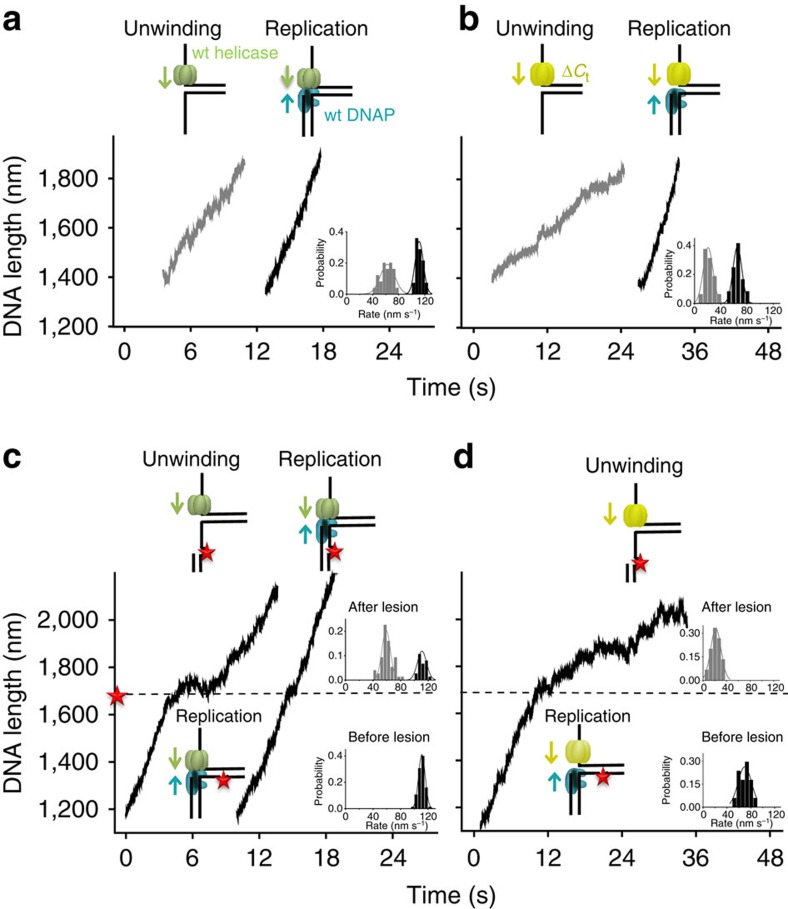
Single-molecule experiments of leading-strand synthesis on a DNA template containing a CPD lesion in the presence of helicase. (**a**,**b**) Helicase unwinding and helicase/DNAP-coupled leading-strand replication on an unmodified DNA template. The helicase's and DNAP's activities were monitored as changes in the DNA length. Representative traces showing DNA length versus time for wt (**a**) or Δ*C*_t_ (**b**) helicase unwinding and a wt DNAP with wt (**a**) or Δ*C*_t_ (**b**) helicase replication in the presence of 0.5 mM dNTP (each) under 6 pN. For clarity, traces have been shifted along the time axis. Cartoons illustrate different protein compositions at the fork for each trace. Insets display distributions of DNA length increase rates for each condition. (**c**,**d**) Helicase/DNAP-coupled leading-strand replication on a DNA template containing a single CPD lesion (red star). Representative traces showing DNA length versus time for a wt DNAP with either a wt helicase (**c**) or a Δ*C*_t_ helicase (**d**) in the presence of 0.5 mM dNTP (each) under 6 pN. The dotted lines indicate the position of a single CPD lesion. For clarity, traces have been shifted along the time axis. Cartoons illustrate different protein compositions at the fork before and after the lesion for each trace. Insets display the distributions of DNA length increase rates before and after the lesion.

In the replisome, T7 DNAP and helicase work synergistically to increase the activities of DNA unwinding and synthesis during leading-strand replication^21^,^25^. The DNAP stimulates helicase unwinding, while the helicase unwinding allows the DNAP to synthesize processively. This synergy is mediated via direct interactions between the two enzymes in the T7 replisome^26^,^27^,^28^. Since T7 helicase is able to unwind through a leading-strand CPD lesion (Fig. 1a), we reasoned that forward translocation of the helicase may assist wt DNAP in overcoming the lesion via their direct interactions. To test this possibility, we performed single-molecule experiments of leading-strand DNA replication with both wt DNAP and helicase (Supplementary Fig. 1d). To avoid strand displacement synthesis by wt DNAP alone, these experiments were performed under low force of 6 pN under which wt DNAP alone would lead to a decrease in DNA length because of its exonuclease activity (Fig. 2b). We first compared the DNA length increases on an unmodified DNA template in the presence of either helicase alone or helicase–DNAP together. The unwinding by helicase alone resulted in a DNA length increase at a rate of 63±22 nm s^−1^ (mean±s.d.; Fig. 3a). When DNAP was present together with helicase, a DNA length increase of 111±13 nm s^−1^ was observed (Fig. 3a), indicating efficient DNA synthesis with the assistance of helicase. Thus, the DNA length increase rate provides a unique signature that distinguishes movements of DNAP alone, helicase alone and DNAP–helicase together at the fork junction (Figs 2b and 3a).

We then investigated whether wt DNAP was able to synthesize through a lesion on the leading strand in the presence of helicase. Before reaching the lesion, DNAP synthesized DNA efficiently as it travelled with helicase, with a DNA length increase at 109±8 nm s^−1^ (Fig. 3c). After reaching the lesion, 72% of traces showed DNA length increase at a slower rate of 59±12 nm s^−1^ (Fig. 3c), comparable to that of helicase unwinding alone (63±22 nm s^−1^), indicating that helicase continued unwinding without DNA synthesis. This occurred either without detectable pausing (2/3) or with a short pause (1/3; 2.0±1.0 s) at the lesion (Fig. 3c). The observation of continuous helicase unwinding without DNA synthesis beyond a lesion has been reported for the bacteriophage T4 and *Escherichia coli* replisomes^4^,^6^,^7^. However, we were surprised to also find that the remaining 28% of traces showed continued synthesis at high rates (110±13 nm s^−1^) either without (3/4) or with a short pause (1/4; 1.0±0.5 s) at the lesion (Fig. 3c). This finding indicates that a helicase-coupled wt DNAP is, in fact, capable of overcoming the CPD lesion. In this process, T7 helicase stays with the paused DNAP at the lesion and then proceeds with the DNAP through the lesion.

### DNAP–helicase interactions lead to lesion tolerance

To further examine whether this lesion tolerance by wt T7 DNAP is due to its interactions with T7 helicase, we utilized a mutant T7 helicase that lacks 17 carboxyl-terminal amino-acid residues (Δ*C*_t_), which are required for interaction with T7 DNAP^29^. The Δ*C*_t_ mutant of T7 helicase has helicase unwinding activities (Fig. 3b), but does not form a stable complex with the DNAP^29^. The wt helicase was replaced with Δ*C*_t_ mutant in the leading-strand DNA replication assay. Control experiments verified that the DNA length increase rate can still serve as a signal for distinguishing between Δ*C*_t_ helicase and DNAP–Δ*C*_t_ together at the fork junction: Δ*C*_t_ mutant (21±15 nm s^−1^) alone was slower than DNAP–Δ*C*_t_ (65±14 nm s^−1^; Fig. 3b). When DNA synthesis was carried out on a leading strand containing a lesion in the presence of Δ*C*_t_ mutant, an average rate of 70±23 nm s^−1^ was observed before encountering the lesion (Fig. 3d). However, a sudden decrease in the rate to 22±18 nm s^−1^ was observed after the change in lesion position in all traces (Fig. 3d), indicating that on DNAP stalling at the lesion Δ*C*_t_ helicase continued to unwind DNA on its own. Unlike that seen with a wt helicase (Fig. 3c), wt T7 DNAP could never overcome the lesion in the presence of the Δ*C*_t_ helicase mutant. This result highlights that DNAP interactions with the helicase are essential for direct replication through the CPD.

### Ensemble studies confirm the lesion tolerance of T7 replisome

**Figure 4:**
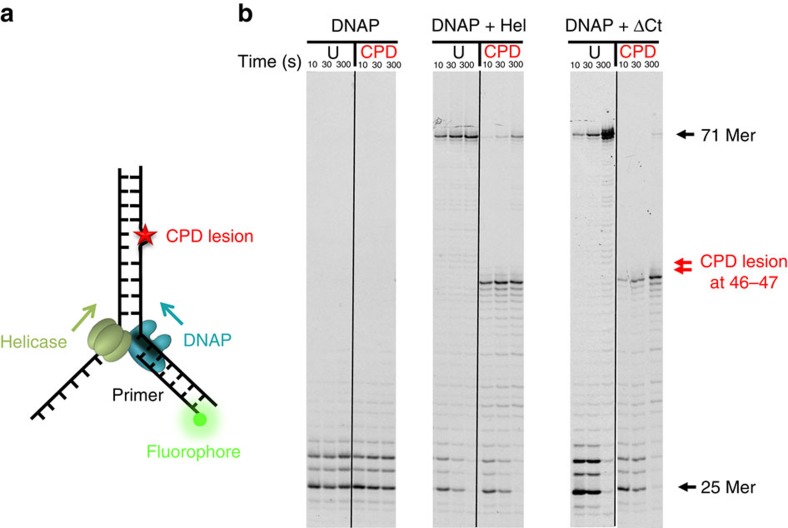
Bulk experiments on DNA synthesis through a lesion-containing DNA template in the presence of helicase. (**a**) Schematic representation of ensemble studies of helicase/DNAP-coupled leading-strand replication on a DNA template containing a single CPD lesion. A CPD lesion (red star) is located at nucleotides 46–47 from 3′ of the template. (**b**) DNA synthesis was carried out by DNAP alone, DNAP with helicase or DNAP with the Δ*C*_t_ mutant helicase. Sequencing gels show the kinetics of the leading-strand DNA synthesis on either an unmodified DNA fork template (denoted as ‘U') or a DNA fork template containing a single CPD lesion (denoted as ‘CPD').

To exclude the possibility that the leading-strand lesion tolerance of T7 replication is due to the tension on the DNA in our single-molecule assays, we carried out a series of ensemble leading-strand DNA replication experiments with single-nucleotide resolution. A replication fork with a 5′ fluorescein-labelled DNA primer was utilized (Fig. 4a). Control experiments verified that, although wt DNAP is incapable of performing strand displacement synthesis by itself, when coupled with wt helicase, it completed the leading-strand synthesis on an unmodified 71-mer DNA template within 10 s (Fig. 4b). On a lesion-containing template, 32% of the helicase-coupled wt DNAP synthesis stalled at the 45 position, just before the lesion at 46–47 positions, and 15% extended to the expected full-length product within 300 s (Fig. 4b). As a shorter synthesis product is expected if wt DNAP bypasses the CPD lesion via re-initiation or frameshift, the full-length product suggests that the wt DNAP has copied the lesion and reinforces the conclusion of direct synthesis through the lesion by DNAP in the presence of helicase. Conversely, in the presence of Δ*C*_t_ helicase, DNAP became blocked at the CPD lesion, and only a negligible amount (3%) showed fully extended primers in 300 s (Fig. 4b). These results again demonstrate that direct interactions between helicase and DNAP assist in the synthesis through a lesion. Similar experiments were also conducted with exo^−^ DNAP, and, in this case, 34% of the primers were extended to full-length products through the lesion in the presence of wt helicase within 300 s (Supplementary Fig. 5). The *E. coli* replisome is able to replicate beyond a leading-strand template lesion by re-priming downstream from the damage^11^. However, our data could not be interpreted in terms of this mechanism in that no ATP or CTP was provided in our assay for priming. In addition, the full-length products clearly indicate fully synthesized DNA without a gap. Therefore, we attribute the lesion-overcoming behaviour to wt DNAP coupled with helicase, directly synthesizing through the lesion and continuing replication.

## Discussion

In this work, we examined the ability of helicase-coupled T7 DNAP to overcome a leading-strand CPD lesion. In isolation, T7 helicase is capable of unwinding a CPD lesion-containing DNA template without detectable pausing or stall. T7 helicase consists of six identical subunits, almost all of which coordinate with the DNA binding/release during unwinding^19^. As only two nucleotides are modified for the CPD lesion template, the multiple binding sites of helicase to DNA may facilitate T7 helicase unwinding through the CPD lesion. In contrast, wt T7 DNAP by itself stalls at the CPD lesion position in both primer extension and strand displacement assays. Crystal structures of T7 DNAP, in complex with DNA and nucleotide substrates, have revealed that T7 DNAP can only accommodate a Watson–Crick base pair via geometric selection^30^. A CPD lesion that prevents the formation of a kink in the DNA backbone would not be readily accommodated by the DNAP's DNA-binding pocket^31^, potentially leading to DNAP stalling at the lesion or synthesis-excision idling, preventing T7 DNAP from replicating through the lesion. Interestingly, we found that once wt T7 DNAP was coupled with T7 helicase, it had the ability to directly synthesize through a CPD lesion because of the interaction between the two enzymes. Because bacteriophage T7 lacks translesion polymerases to perform tranlesion synthesis and, unlike *E. coli*^11^, cannot reinitiate synthesis by re-priming the leading strand^32^, the direct pathway we proposed here is essential for the T7 replisome to tolerate lesions during replication.

The mechanism of the lesion bypass by exo^−^ DNAP might provide some insights on understanding how helicase assists DNAP in overcoming a lesion. Biochemical and crystallographic studies have revealed that the CPD template is excluded from the active site of an exo^−^ DNAP during incorporation of a nucleotide opposite the CPD lesion^24^,^31^,^33^,^34^. It is possible that an intermediate synthesis product generated by wt T7 DNAP, as it attempts to bypass the CPD lesion, is recognized as an incorrect nucleotide because the incorporated nucleotide is not properly paired and is therefore excised by the exonuclease domain so that no bypass occurs. Interestingly, a recent biochemical study revealed that T7 helicase is positioned one nucleotide ahead of T7 DNAP at the fork during leading-strand synthesis and stabilizes the post-translocation state of the DNAP^35^. This may impair T7 DNAP's switch from the polymerase state to the exonuclease state as its exonuclease activity requires the DNAP to be in the pre-translocation state first^36^. Our results also indicate that the association of DNAP with helicase suppresses the exonuclease activity of DNAP (Figs 2b and 3a). Such an association may alleviate the regression pressure from re-annealing fork (Fig. 3a) to allow DNAP to overcome the lesion as was seen with exo^−^ DNAP alone. However, we believe the T7 helicase plays a larger role in facilitating DNAP overcoming lesions because a wt DNAP alone was unable to overcome the lesion in primer extension assays where fork re-annealing was absent (Fig. 2d and Supplementary Fig. 4). Because interactions between T7 DNAP and helicase are essential for CPD lesion tolerance, it is possible that T7 helicase keeps the polymerase tethered to its substrate via these interactions, allowing for multiple rounds of attempted synthesis over the lesion, and thus increasing the chance of lesion bypass. The details are yet to be fully elucidated.

It has long been believed that leading-strand DNA lesions are major obstacles for replication progression, as the high-fidelity replicative polymerase is unable to directly proceed through them. For example, in both the bacteriophage T4 and *E. coli* replisomes, it is concluded that the leading- and lagging-strand DNA replication becomes uncoupled after the replisome encounters a leading-strand DNA lesion, with leading-strand synthesis stalling and helicase unwinding continuing^4^,^6^,^7^. A few indirect pathways have been demonstrated to explain how a stalled replication fork could circumvent DNA lesions to restart the replication^2^. These lesion bypass pathways, however, require the replisome to avoid lesions and/or reinitiate replication after bypass^2^. On the basis of our studies, we propose a new lesion tolerance pathway for leading-strand synthesis in which a replicative DNAP directly synthesizes through a leading-strand lesion with the assistance of helicase. In this pathway, helicase and DNAP are functionally coupled to unwind dsDNA and synthesize the nascent DNA. DNAP stalls after it encounters a lesion in the leading-strand template; however, helicase is unaffected by the lesion, but will transiently stall because of its interaction with DNAP. Ultimately, the forward motion of the helicase may assist the paused DNAP in passing through the lesion by means of the interactions between them. This new pathway may be particularly essential for viruses that lack specialized proteins for other lesion bypass pathways. In addition, in contrast to other pathways that might result in replication fork delay due to the recruitment of other accessory proteins and/or the re-initiation of the replisome, this pathway is more efficient in terms of replisome recovery and the absence of ssDNA gaps. These characteristics would be particularly advantageous when replication needs to be completed in a timely manner.

## Methods

### Preparation of proteins and DNA

The untagged proteins, T7 gp4, gp4A′ and delta C gp4A′ mutant, were overexpressed in the BL21 DE3 cell line and T7 gp5 (D5A and D7A) was expressed in the A179 cell line containing pGP1 plasmid. The cells were lysed by three freeze thaw cycles in 20 mM phosphate buffer pH 7.4, with 50 mM NaCl, 10% glycerol, 2 mM dithiothreitol (DTT), 2 mM beta mercaptoethanol and 1 or 2.5 mM EDTA (for helicase or gp5, respectively) in the presence of 0.2 mg ml^−1^ lysozyme. Polyethyleneimine precipitation was carried out by increasing the salt concentration to 0.5 M. Supernatant was precipitated in 70% ammonium sulfate and purified with Phosphocellulose (P11 resin) followed by DEAE Sepharose column chromatography^29^,^37^,^38^. *E. coli* thioredoxin (trx) was purchased from Sigma-Aldrich (St Louis, MO). wt T7 DNAP was purchased from New England Biolabs (NEB, Ipswich, MA). Primers and unmodified oligonucleotides were purchased from Integrated DNA Technologies (IDT, San Diego, CA). *Cis-syn* thymine dimer phosphoramidite was purchased from Glen Research (Sterling, VA). The CPD-containing DNA oligonucleotides were synthesized and purified using PAGE electrophoresis by Oligos Etc (Wilsonville, OR).

The DNA template used for single-molecule experiments consisted of three pieces: two arms and the trunk (Supplementary Fig. 1a)^39^. Arm 1 (1,129 bp) was amplified from plasmid pLB574 (ref. 40) using a digoxigenin-labelled primer. Resulting DNA fragments were digested with BstXI (NEB) to create an overhang and were subsequently annealed to a short DNA with a complementary overhang formed by adapter 1 (5′-/phos/GCAGTACCGAGCTCATCCAATTCTACATGCCGC-3′) and adapter 2 (5′-/phos/GCCTTGCACGTGATTACGAGATATCGATGATTGCGGCGGCATGTAGAATTGGATGAGCTCGGTACTGCATCG-3′). Arm 2 (2,013 bp) was amplified from plasmid pBR322 (NEB) using a biotin-labelled primer. Resulting DNA fragments were digested with BstEII (NEB) to create an overhang and were subsequently annealed to adapter 3 (5′-/phos/GTAACCTGTACAGTGTATAGAATGACGTAACGCGCAATCATCGATATCTCGTAATCACGTGCAAGGCCTA-3′). The adapter 3 from arm 2 and the adapter 2 from arm 1 were partially complementary to each other and were annealed to create a short ∼30-bp trunk with a 3-bp overhang for the trunk ligation. The trunk was a ligation product of a three-piece DNA segment: a 1.1-kbp upstream segment, a 71-bp lesion segment and a 1.1-kbp downstream segment. The upstream segment was made via PCR from plasmid generated based on pRL574 (ref. 41) and BsaI (NEB) to create overhangs for ligation with the lesion segment. The lesion segment was made by annealing of two oligos (5′-/phos/GGTGTCACCAGCAGGCCGATTGGG**TT**GGGTATTCGCCGTGTCCCTCTCGATGGCTGTAAGTATCCTATAGG-3′ and 5′-/phos/ACCGCCTATAGGATACTTACAGCCATCGAGAGGGACACGGCGAATACCCAACCCAATCGGCCTGCTGGTGACACCCGAT-3′). The downstream segment was made via PCR from plasmid generated based on pRL574 (ref. 41) and digested with BstXI (NEB) to create an overhang for ligation with the lesion segment. These three pieces of DNA segments were ligated and purified. On the day of an experiment, the arms and the trunk were mixed and ligated at 16 °C for 3 h.

To create the DNA template with a CPD lesion located on the leading-strand template, the upstream DNA segment was digested with AlwNI, before ligating with the lesion segment, to create overhangs for ligation with the 30-bp trunk of the arms, resulting in a CPD lesion located at 1,145–1,146 bp from the initial fork. To create the DNA template with a CPD lesion located on the lagging-strand template, the trunk was flipped and the downstream DNA segment, instead of the upstream DNA segment, was digested with AlwNI to create overhangs for ligation with the arms, resulting in a CPD lesion located at 1,223–1,224 bp from the initial fork (Supplementary Fig. 1a).

### Single-molecule assays

Sample chambers were prepared as follow^18^,^19^. Briefly, DNA tethers were formed by first nonspecifically coating the sample chamber surface with antidigoxigenin (Roche, Indianapolis, IN), which binds nonspecifically to the coverglass surface, followed by an incubation with digoxigenin-tagged DNA. Streptavidin-coated 0.48-mm polystyrene microspheres (Polysciences, Warrington, PA) were then added to the chamber. Finally, the protein solution was flowed into the sample chamber just before data acquisition. The replication buffer consisted of 50 mM Tris-HCl (pH 7.5), 40 mM NaCl, 10% glycerol, 1.5 mM EDTA, 2 mM DTT and dNTPs at the concentrations specified in the text, and MgCl_2_ at a concentration of 2.5 mM in excess of the total nucleotide concentration. Experiments were conducted in a climate-controlled room at a temperature of 23.3 °C; however, owing to local laser trap heating the temperature increased slightly to 25±1 °C (ref. 42).

Helicase unwinding and polymerase strand displacement experiments were conducted as follows. First, several hundred base pairs of dsDNA were mechanically unzipped (with an average unzipping force ∼15 pN), at a constant velocity of 1,400 bp s^−1^ to produce a ssDNA loading region for helicase or a priming template for polymerase. Second, DNA length was maintained until a force drop below a threshold, indicating helicase or polymerase unwinding of the DNA fork. Finally, a constant force was maintained while helicase or polymerase unwound the dsDNA.

For the helicase-only experiments, to ensure that only one helicase was loaded on the template in the helicase unwinding assay, we used a low helicase concentration (0.4 nM hexamer) so that the average helicase-loading time was significantly longer than the typical ssDNA translocation and dsDNA-unwinding time^18^.

For the DNAP-only experiments, Exo^−^ DNAP was assembled by adding 10 μM of gp5 in 50 μM *E. coli* trx and incubating at room temperature for 5 min. wt DNAP from NEB contains gp5 and trx and was used directly. Before data acquisition, 30 nM of the appropriate DNAP in 50 μl replication buffer was flowed into the chamber.

DNA synthesis in the presence of helicase was performed by maintaining a constant force at 6 pN. The helicase and polymerase were added as follows: first, 30 nM of the appropriate helicase hexamer was incubated for 10 min in the replication buffer on ice, then 30 nM of the appropriate DNAP was added and the solution was incubated for 10 min at room temperature. The resulting 50-μl solution was flowed into the chamber just before data acquisition. The DNA template designed with a 27-nt initial ssDNA region (Supplementary Fig. 1a) accommodates only one helicase with one DNAP loading at the fork as each T7 helicase has been shown to bind and protect 25–30 bases of ssDNA^43^,^44^,^45^. These low concentrations of helicase and DNAP added at a stoichiometric ratio ensured that the experiments were most likely conducted under a single-molecule condition. On very rare occasions, the DNA length signal remained constant at the lesion position for 2 min (experimental cutoff time), suggesting that helicase, together with DNAP, was stalled by the lesion because of the interaction between them.

Considering that non-coupled DNAP may alter the helicase unwinding rate, the wt helicase and Δ*C*_t_ unwinding rates in Fig. 3a,b were measured in the presence of DNAP using a modified template with a 3′ inverted dT incorporated at the adapter 1 (IDT) from which DNAP could not synthesize.

### Data collection and analysis

Data were low-pass-filtered to 5 kHz and digitized at 12 kHz, and then were further averaged to 110 Hz. The acquired data signals were converted into force and DNA extension as previously described^18^. Elasticity parameters of both dsDNA and ssDNA were necessary for data conversion and were obtained from the DNA force-extension measurements. The force-extension relation of dsDNA was described using a modified Marko Siggia worm-like-chain model^46^: the contour length per base was 0.338 nm, the persistence length of DNA was 44.5 nm and the stretch modulus was 1,200 pN. The force-extension relation of ssDNA was described using an extensible freely jointed-chain model^47^: for forces higher than 12 pN, a counter length per base was 0.52 nm, Kuhn length was 1.91 nm and a stretch modulus was 393 pN. For forces lower than 12 pN, the force extension was directly determined using a helicase-based method as previously described^18^. For the helicase unwinding studies, one base pair unwound generated two nucleotides of ssDNA. Accordingly, real-time DNA extension was converted into the number of base pairs unwound. To improve positional accuracy and precision, the data were then aligned to a theoretical unzipping curve for the mechanically unzipped section of the DNA^48^,^49^. For the DNAP strand displacement synthesis studies, one separated base pair was converted to one base pair of dsDNA via DNA synthesis and one nucleotide of ssDNA. Accordingly, DNA extension was converted into the number of nucleotides synthesized or excised by DNAP.

For the single-molecule replication assay with a lesion-containing template in Fig. 3, we had to first determine whether the movement was due to helicase alone, or DNAP synthesis coupled with helicase unwinding before and after the lesion, and this was more readily achieved by directly measuring the DNA length increase rates in nm s^−1^. Therefore, we presented data as DNA length in nm and rates in nm s^−1^. Under 6 pN of force used, 1 nm increase in length corresponded to 1.95 bp unwounded by helicase and 1.74 bp replicated by the leading-strand synthesis via DNAP. As the position of the CPD lesion was known from the DNA template design (1,145–1,146 bp from the initial fork), the lesion position in nm showed in Fig. 3 was determined by converting bp to nm, assuming that the DNA template before the lesion was replicated.

### Ensemble assays

DNA replication and synthesis were measured using a rapid quenched-flow instrument at 18 °C (ref. 50). A primer (5′-/FluorT/CCTATAGGATACTTACAGCCATCGA-3′) and a lesion-containing template (5′-GGTGTCACCAGCAGGCCGATTGGG**TT**GGGTATTCGCCGTGTCCCTCTCGATGGCTGTAAGTATCCTATAGG-3′) were annealed together for the primer extension assay. Replication fork templates for the replication assay were prepared by annealing a primer, a template (both listed above) and a top oligonucleotide together (5′-AACGCCAAGCCAGGTATAAAGCATGGAGGGACACGGCGAAATACCCAACCCAATCGGCCTGCTGGTGACACC-3′). DNAP (65 nM) alone or helicase (65 nM) and DNAP (65 nM) together were preassembled on 50-nM DNA in a buffer containing 50 mM Tris-HCl (pH 7.5), 40 mM NaCl, 10% glycerol and 1 mM dNTP (each). To initiate reactions, 4 mM free MgCl_2_ was added. Reactions were quenched with 150 mM EDTA and subsequently resolved on 24% acrylamide/7 M urea/1.5 × TBE gels.

## Additional information

**How to cite this article**: Sun, B. *et al.* T7 replisome directly overcomes DNA damage. *Nat. Commun.* 6:10260 doi: 10.1038/ncomms10260 (2015).

## Supplementary Material

Supplementary Information Supplementary Figures 1-5 and Supplementary References

## References

[b1] Cox M. M. *et al.* The importance of repairing stalled replication forks. Nature 404, 37–41 (2000). 10716434 10.1038/35003501

[b2] Yeeles J. T. P., Poli J., Marians K. J. & Pasero P. Rescuing stalled or damaged replication forks. Cold Spring Harb. Perspect. Biol. 5, 1–15 (2013). 10.1101/cshperspect.a012815 PMC3632063 23637285

[b3] Kowalczykowski S. C. Initiation of genetic recombination and recombination-dependent replication. Trends Biochem. Sci. 25, 156–165 (2000). 10754547 10.1016/s0968-0004(00)01569-3

[b4] Nelson S. W. & Benkovic S. J. Response of the bacteriophage T4 replisome to noncoding lesions and regression of a stalled replication fork. J. Mol. Biol. 401, 743–756 (2010). 20600127 10.1016/j.jmb.2010.06.027 PMC2943651

[b5] McInerney P. & O'Donnell M. Functional uncoupling of twin polymerases: mechanism of polymerase dissociation from a lagging-strand block. J. Biol. Chem. 279, 21543–21551 (2004). 15014081 10.1074/jbc.M401649200

[b6] McInerney P. & O'Donnell M. Replisome fate upon encountering a leading strand block and clearance from DNA by recombination proteins. J. Biol. Chem. 282, 25903–25916 (2007). 17609212 10.1074/jbc.M703777200

[b7] Higuchi K. *et al.* Fate of DNA replication fork encountering a single DNA lesion during oriC plasmid DNA replication *in vitro*. Genes Cells 8, 437–449 (2003). 12694533 10.1046/j.1365-2443.2003.00646.x

[b8] Zhu B., Lee S. J. & Richardson C. C. Bypass of a nick by the replisome of bacteriophage T7. J. Biol. Chem. 286, 28488–28497 (2011). 21701044 10.1074/jbc.M111.252023 PMC3151091

[b9] Manosas M., Perumal S. K., Croquette V. & Benkovic S. J. Direct observation of stalled fork restart via fork regression in the T4 replication system. Science 338, 1217–1220 (2012). 23197534 10.1126/science.1225437 PMC3858903

[b10] Courcelle J., Donaldson J. R., Chow K. H. & Courcelle C. T. DNA damage-induced replication fork regression and processing in *Escherichia coli*. Science 299, 1064–1067 (2003). 12543983 10.1126/science.1081328

[b11] Yeeles J. T. P. & Marians K. J. The *Escherichia coli* replisome is inherently DNA damage tolerant. Science 334, 235–238 (2011). 21998391 10.1126/science.1209111 PMC3593629

[b12] Sale J. E., Lehmann A. R. & Woodgate R. Y-family DNA polymerases and their role in tolerance of cellular DNA damage. Nat. Rev. Mol. Cell Biol. 13, 141–152 (2012). 22358330 10.1038/nrm3289 PMC3630503

[b13] Kath J. E. *et al.* Polymerase exchange on single DNA molecules reveals processivity clamp control of translesion synthesis. Proc. Natl Acad. Sci. USA 111, 7647–7652 (2014). 24825884 10.1073/pnas.1321076111 PMC4040570

[b14] Rudolph C. J., Upton A. L. & Lloyd R. G. Replication fork stalling and cell cycle arrest in UV-irradiated *Escherichia coli*. Genes Dev. 21, 668–681 (2007). 17369400 10.1101/gad.417607 PMC1820941

[b15] Hamdan S. M. & Richardson C. C. Motors, switches, and contacts in the replisome. Annu. Rev. Biochem. 78, 205–243 (2009). 19298182 10.1146/annurev.biochem.78.072407.103248

[b16] Delagoutte E. & von Hippel P. H. Helicase mechanisms and the coupling of helicases within macromolecular machines - Part II: integration of helicases into cellular processes. Q. Rev. Biophys. 36, 1–69 (2003). 12643042 10.1017/s0033583502003864

[b17] Brown W. C. & Romano L. J. Benzo[a]pyrene-DNA adducts inhibit translocation by the gene 4 protein of bacteriophage T7. J. Biol. Chem. 264, 6748–6754 (1989). 2708341

[b18] Johnson D. S., Bai L., Smith B. Y., Patel S. S. & Wang M. D. Single-molecule studies reveal dynamics of DNA unwinding by the ring-shaped T7 helicase. Cell 129, 1299–1309 (2007). 17604719 10.1016/j.cell.2007.04.038 PMC2699903

[b19] Sun B. *et al.* ATP-induced helicase slippage reveals highly coordinated subunits. Nature 478, 132–135 (2011). 21927003 10.1038/nature10409 PMC3190587

[b20] Tabor S., Huber H. E. & Richardson C. C. *Escherichia coli* thioredoxin confers processivity on the DNA polymerase activity of the gene 5 protein of bacteriophage T7. J. Biol. Chem. 262, 16212–16223 (1987). 3316214

[b21] Stano N. M. *et al.* DNA synthesis provides the driving force to accelerate DNA unwinding by a helicase. Nature 435, 370–373 (2005). 15902262 10.1038/nature03615 PMC1563444

[b22] Manosas M. *et al.* Mechanism of strand displacement synthesis by DNA replicative polymerases. Nucleic Acids Res. 40, 6174–6186 (2012). 22434889 10.1093/nar/gks253 PMC3401438

[b23] McCulloch S. D. & Kunkel T. A. Multiple solutions to inefficient lesion bypass by T7 DNA polyrnerase. DNA Repair (Amst). 5, 1373–1383 (2006). 16876489 10.1016/j.dnarep.2006.06.003 PMC1892196

[b24] Smith C. A., Baeten J. & Taylor J. S. The ability of a variety of polymerases to synthesize past site-specific cis-syn, trans-syn-II, (6-4), and Dewar photoproducts of thymidylyl-(3 '-> 5 ')-thymidine. J. Biol. Chem. 273, 21933–21940 (1998). 9705333 10.1074/jbc.273.34.21933

[b25] Pandey M. & Patel S. S. Helicase and polymerase move together close to the fork junction and copy DNA in one-nucleotide steps. Cell Rep. 6, 1129–1138 (2014). 24630996 10.1016/j.celrep.2014.02.025 PMC4010093

[b26] Kulczyk A. W. *et al.* An interaction between DNA polymerase and helicase is essential for the high processivity of the bacteriophage T7 replisome. J. Biol. Chem. 287, 39050–39060 (2012). 22977246 10.1074/jbc.M112.410647 PMC3493946

[b27] Hamdan S. M. *et al.* Dynamic DNA helicase-DNA polymerase interactions assure processive replication fork movement. Mol. Cell 27, 539–549 (2007). 17707227 10.1016/j.molcel.2007.06.020

[b28] Zhang H. *et al.* Helicase-DNA polymerase interaction is critical to initiate leading-strand DNA synthesis. Proc. Natl Acad. Sci. USA 108, 9372–9377 (2011). 21606333 10.1073/pnas.1106678108 PMC3111293

[b29] Notarnicola S. M., Mulcahy H. L., Lee J. & Richardson C. C. The acidic carboxyl terminus of the bacteriophage T7 gene 4 helicase/primase interacts with T7 DNA polymerase. J. Biol. Chem. 272, 18425–18433 (1997). 9218486 10.1074/jbc.272.29.18425

[b30] Doublie S., Tabor S., Long A. M., Richardson C. C. & Ellenberger T. Crystal structure of a bacteriophage T7 DNA replication complex at 2.2 angstrom resolution. Nature 391, 251–258 (1998). 9440688 10.1038/34593

[b31] Li Y. *et al.* Nucleotide insertion opposite a cis-syn thymine dimer by a replicative DNA polymerase from bacteriophage T7. Nat. Struct. Mol. Biol. 11, 784–790 (2004). 15235589 10.1038/nsmb792

[b32] Romano L. J., Tamanoi F. & Richardson C. C. Initiation of DNA replication at the primary origin of bacteriophage T7 by purified proteins: requirement for T7 RNA polymerase. Proc. Natl Acad. Sci. USA 78, 4107–4111 (1981). 6945573 10.1073/pnas.78.7.4107 PMC319735

[b33] Sun L. P., Wang M., Kool E. T. & Taylor J. S. Pyrene nucleotide as a mechanistic probe: evidence for a transient abasic site-like intermediate in the bypass of dipyrimidine photoproducts by T7 DNA polymerase. Biochemistry 39, 14603–14610 (2000). 11087416 10.1021/bi001446v

[b34] Taylor J. S. New structural and mechanistic insight into the A-rule and the instructional and non-instructional behavior of DNA photoproducts and other lesions. Mutat. Res. 510, 55–70 (2002). 12459443 10.1016/s0027-5107(02)00252-x

[b35] Nandakumar D., Pandey M. & Patel S. S. Cooperative base pair melting by helicase and polymerase positioned one nucleotide from each other. Elife 4, 1–23 (2015). 10.7554/eLife.06562 PMC4460406 25970034

[b36] Lieberman K. R., Dahl J. M. & Wang H. Kinetic mechanism at the branchpoint between the DNA synthesis and editing pathways in individual DNA polymerase complexes. J. Am. Chem. Soc. 136, 7117–7131 (2014). 24761828 10.1021/ja5026408 PMC4046759

[b37] Patel S. S., Rosenberg A. H., Studier F. W. & Johnson K. A. Large scale purification and biochemical characterization of T7 primase/helicase proteins. Evidence for homodimer and heterodimer formation. J. Biol. Chem. 267, 15013–15021 (1992). 1321824

[b38] Patel S. S., Wong I. & Johnson K. A. Pre-steady-state kinetic analysis of processive DNA replication including complete characterization of an exonuclease-deficient mutant. Biochemistry 30, 511–525 (1991). 1846298 10.1021/bi00216a029

[b39] Inman J. T. *et al.* DNA Y structure: a versatile, multidimensional single molecule assay. Nano Lett. 14, 6475–6480 (2014). 25291441 10.1021/nl503009d PMC4245981

[b40] Schafer D. A., Gelles J., Sheetz M. P. & Landick R. Transcription by single molecules of rna-polymerase observed by light-microscopy. Nature 352, 444–448 (1991). 1861724 10.1038/352444a0

[b41] Jin J. *et al.* Synergistic action of RNA polymerases in overcoming the nucleosomal barrier. Nat. Struct. Mol. Biol. 17, 745–752 (2010). 20453861 10.1038/nsmb.1798 PMC2938954

[b42] Peterman E. J., Gittes F. & Schmidt C. F. Laser-induced heating in optical traps. Biophys. J. 84, 1308–1316 (2003). 12547811 10.1016/S0006-3495(03)74946-7 PMC1302707

[b43] Hingorani M. M. & Patel S. S. Interactions of bacteriophage T7 DNA primase/helicase protein with single-stranded and double-stranded DNAs. Biochemistry 32, 12478–12487 (1993). 8241139 10.1021/bi00097a028

[b44] Egelman E. H., Yu X., Wild R., Hingorani M. M. & Patel S. S. Bacteriophage T7 helicase/primase proteins form rings around single-stranded DNA that suggest a general structure for hexameric helicases. Proc. Natl Acad. Sci. USA 92, 3869–3873 (1995). 7731998 10.1073/pnas.92.9.3869 PMC42063

[b45] Patel S. S. & Hingorani M. M. Oligomeric structure of bacteriophage T7 DNA primase/helicase proteins. J. Biol. Chem. 268, 10668–10675 (1993). 8486715

[b46] Wang M. D., Yin H., Landick R., Gelles J. & Block S. M. Stretching DNA with optical tweezers. Biophys. J. 72, 1335–1346 (1997). 9138579 10.1016/S0006-3495(97)78780-0 PMC1184516

[b47] Smith S. B., Cui Y. & Bustamante C. Overstretching B-DNA: the elastic response of individual double-stranded and single-stranded DNA molecules. Science 271, 795–799 (1996). 8628994 10.1126/science.271.5250.795

[b48] Hall M. A. *et al.* High-resolution dynamic mapping of histone-DNA interactions in a nucleosome. Nat. Struct. Mol. Biol. 16, 124–129 (2009). 19136959 10.1038/nsmb.1526 PMC2635915

[b49] Shundrovsky A., Smith C. L., Lis J. T., Peterson C. L. & Wang M. D. Probing SWI/SNF remodeling of the nucleosome by unzipping single DNA molecules. Nat. Struct. Mol. Biol. 13, 549–554 (2006). 16732285 10.1038/nsmb1102

[b50] Pandey M., Levin M. K. & Patel S. S. Experimental and computational analysis of DNA unwinding and polymerization kinetics. Methods. Mol. Biol. 587, 57–83 (2010). 20225142 10.1007/978-1-60327-355-8_5 PMC3787510

