# Supplementary material for: T7 replisome directly overcomes DNA damage
Source: Nat Commun. 2015 Dec 17;6:10260. doi: 10.1038/ncomms10260 (PMC4703881; doi:10.1038/ncomms10260)
Supplement: Supplementary Information — Supplementary Figures 1-5 and Supplementary References [file ncomms10260-s1.pdf]

Supplementary Figures

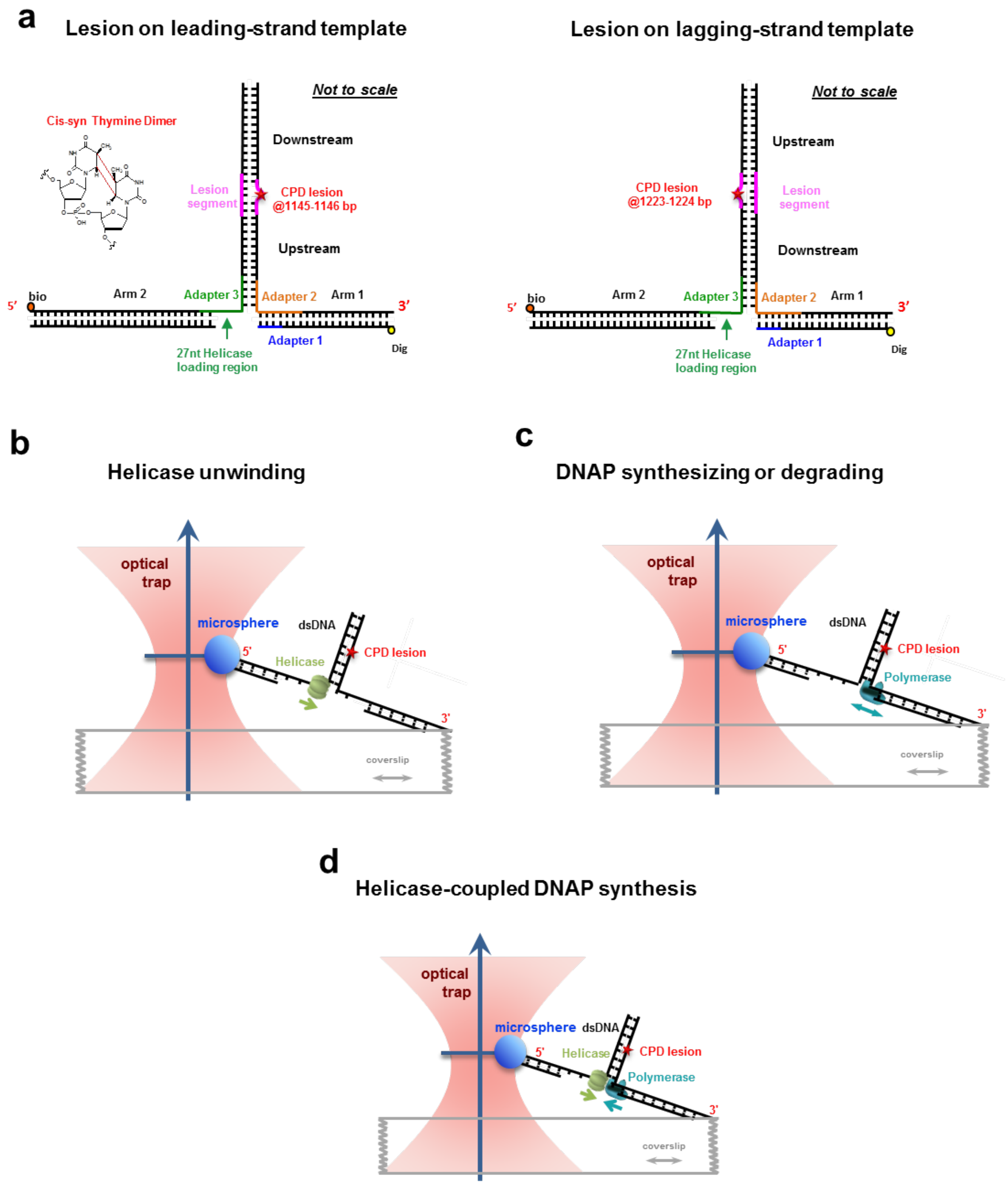

Supplementary Figure 1: DNA design and experimental configuration.

a, DNA template design for single-molecule assays. The DNA construct was made from three DNA segments: two arms and trunk, linked through three short adapters[1] (see Methods). The digoxigenin (dig) and biotin labels were located at the ends of two arms for binding to the coverglass and microsphere, respectively. The CPD lesion (chemical structure shown in inset and denoted as red star in DNA construct) segment located in the trunk was ligated to upstream and downstream DNA segments. The DNA construct with a lesion located on the leading-strand template was made by ligating the upstream DNA segment with the arms after digestion with AlwNI, resulting in the CPD lesion located at 1145-1146 bp from the fork, and the DNA construct with a lesion located on the lagging-strand temple was made by flipping the trunk and ligating the downstream DNA segment with the arms after digestion with AlwNI, resulting in the CPD lesion located at 1223-1224 bp from the fork. The 27 nt ssDNA on adapter 3 allows helicase to bind and unwind the trunk. The DNA polymerase starts to synthesize from the 3' of adapter 1.

b, c, d, Single-molecule experimental configurations for the observation of helicase unwinding (**b**), DNA polymerase (DNAP) synthesizing (or degrading) DNA (**c**), and the helicase-unwinding coupled leading strand DNA synthesis by DNAP (**d**). One arm of the DNA construct was attached to a trapped microsphere via a biotin/streptavidin connection. The other arm was anchored to a microscope coverslip surface via a dig/antidig connection. The trapped microsphere was held in a feedback-enhanced optical trap so that its position relative to the trap center and the trapping force could be measured. Helicase unwinding (**b**), DNA synthesis or degradation (**c**), and helicase-unwinding coupled DNA synthesis (**d**) was monitored as a change in the DNA length.

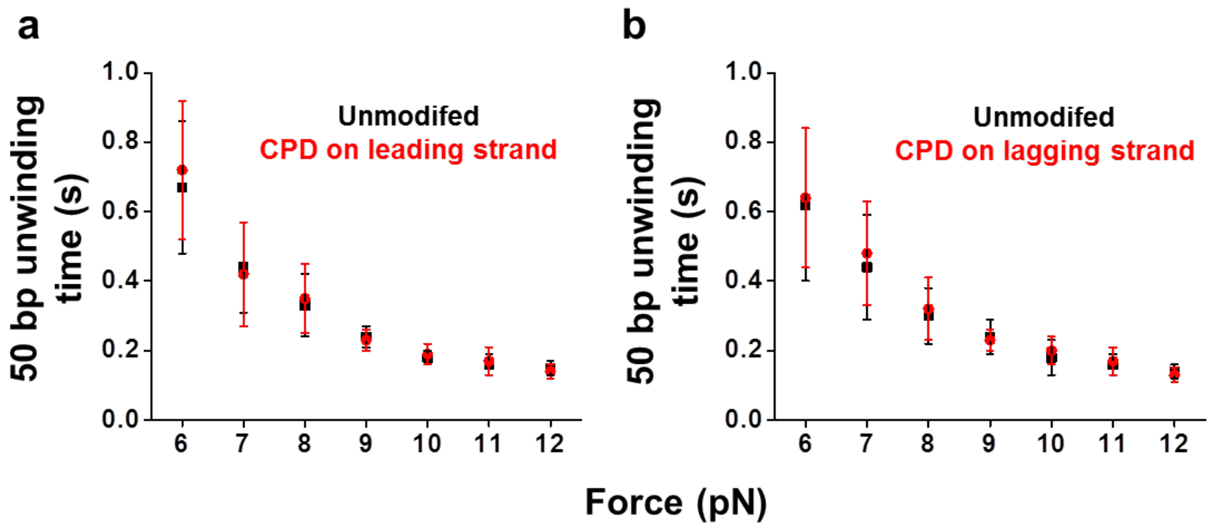

Supplementary Figure 2: Helicase unwinding time of dsDNA with or without a CPD lesion.

a, b, Comparison of helicase unwinding time of 50 bp dsDNA containing no lesions or a single CPD lesion on either leading strand (**a**) or lagging strand (**b**). To determine the unwinding time, each unwinding trace was first smoothed to 5 Hz. These data show that the force-dependent unwinding time for a CPD-containing template on either leading or lagging strand is indistinguishable from those for unmodified dsDNA, indicating that helicase unwound a CPD lesion-containing template without detectable stalls or pauses.

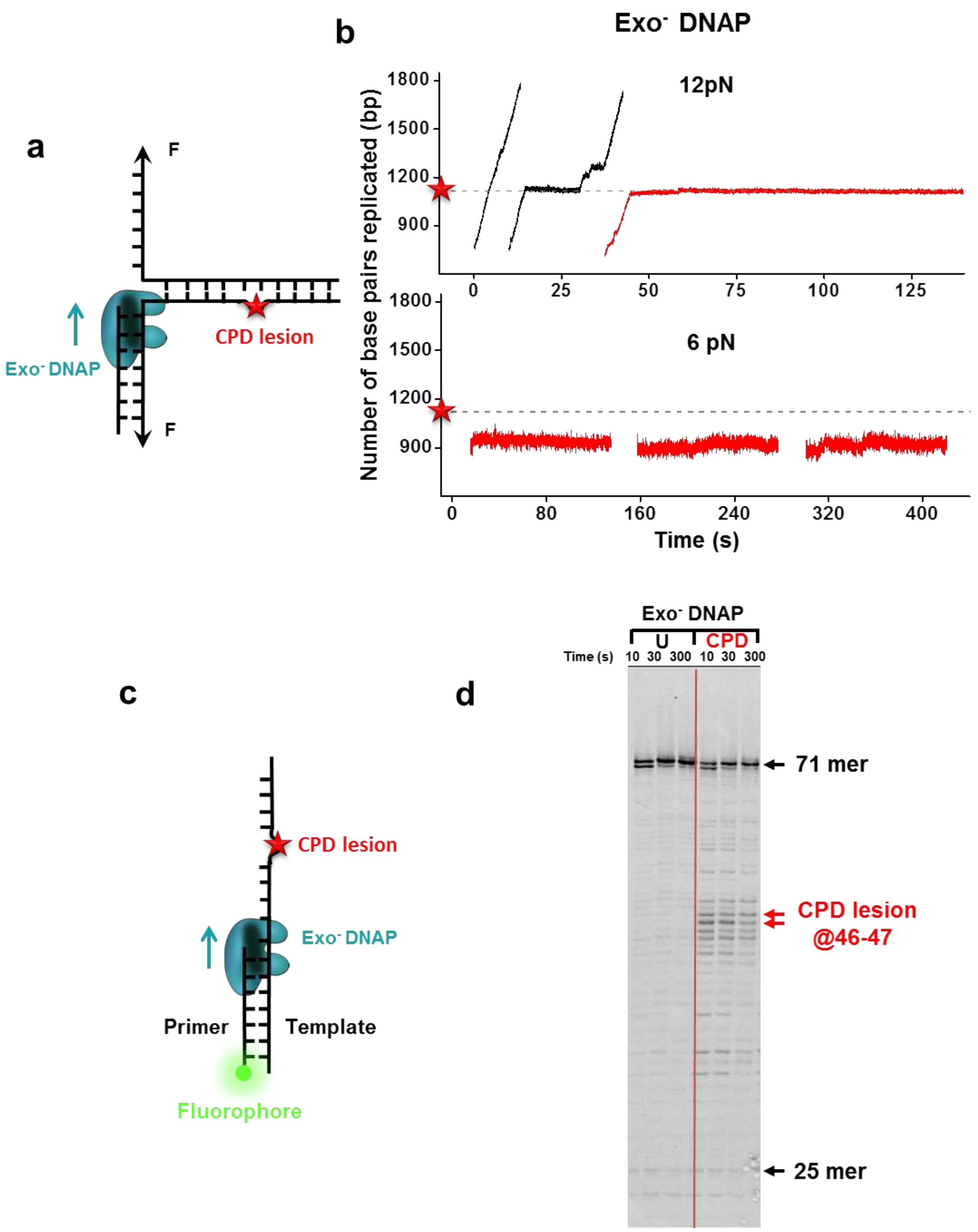

Supplementary Figure 3: Exo$^-$ T7 DNAP synthesizes through a CPD lesion.

a, Schematic representation of the single-molecule configuration of T7 DNAP synthesis on a template containing a CPD lesion. Two arms of the template were hold at a constant unzipping

force of 12 pN or 6 pN while T7 DNAP synthesized from the 3’ of the primer. The CPD lesion was located in the template strand and denoted as a red star.

b, Representative traces showing the number of base pairs replicated versus time in the presence of 1 mM dNTPs under 12 pN or 6 pN for exo$^-$ T7 DNAP. We found that, under 12 pN, 65% of traces replicated through the lesion (e.g., black traces), without any detectable pausing (3/4) or with a short pause (1/4; 6.4 ± 4.5 s) at the lesion before proceeding through, while the remaining traces (e.g., red trace) indicated exo$^-$ T7 DNAP stalled at the lesion for at least two minutes (experimental cutoff time). However, exo$^-$ T7 DNAP was incapable of performing strand displacement synthesis under 6 pN. In contrast to the wt DNAP (Fig. 2b), we did not observe a decrease in DNA extension under this force. These results suggest that a decrease in DNA extension with wt DNAP under 6 pN was due to its exonuclease activity (Fig. 2b). The dotted line indicates the lesion position.

c, Schematic representation of the primer extension on a template containing a CPD lesion by exo$^-$ T7 DNAP in ensemble studies. The 25-mer primer with 5' fluorescein labeled was annealed to the template 71-mer which contains a CPD lesion (red star) at 46th and 47th nucleotides from 3’ end of the template.

d, A denaturing PAGE gel analysis of elongation of the primer by exo$^-$ T7 DNAP on the template. Control experiments utilizing an unmodified template (denoted as “U”) showed that the primers were extended to full length by exo$^-$ T7 DNAP in 30 seconds. On a CPD lesion-containing template (denoted as “CPD”), exo$^-$ T7 DNAP paused before and after the lesion but was able to extend 50% of the primers through the lesion (≥ 48 nt). The ensemble results are in reasonable agreement with our single-molecule data.

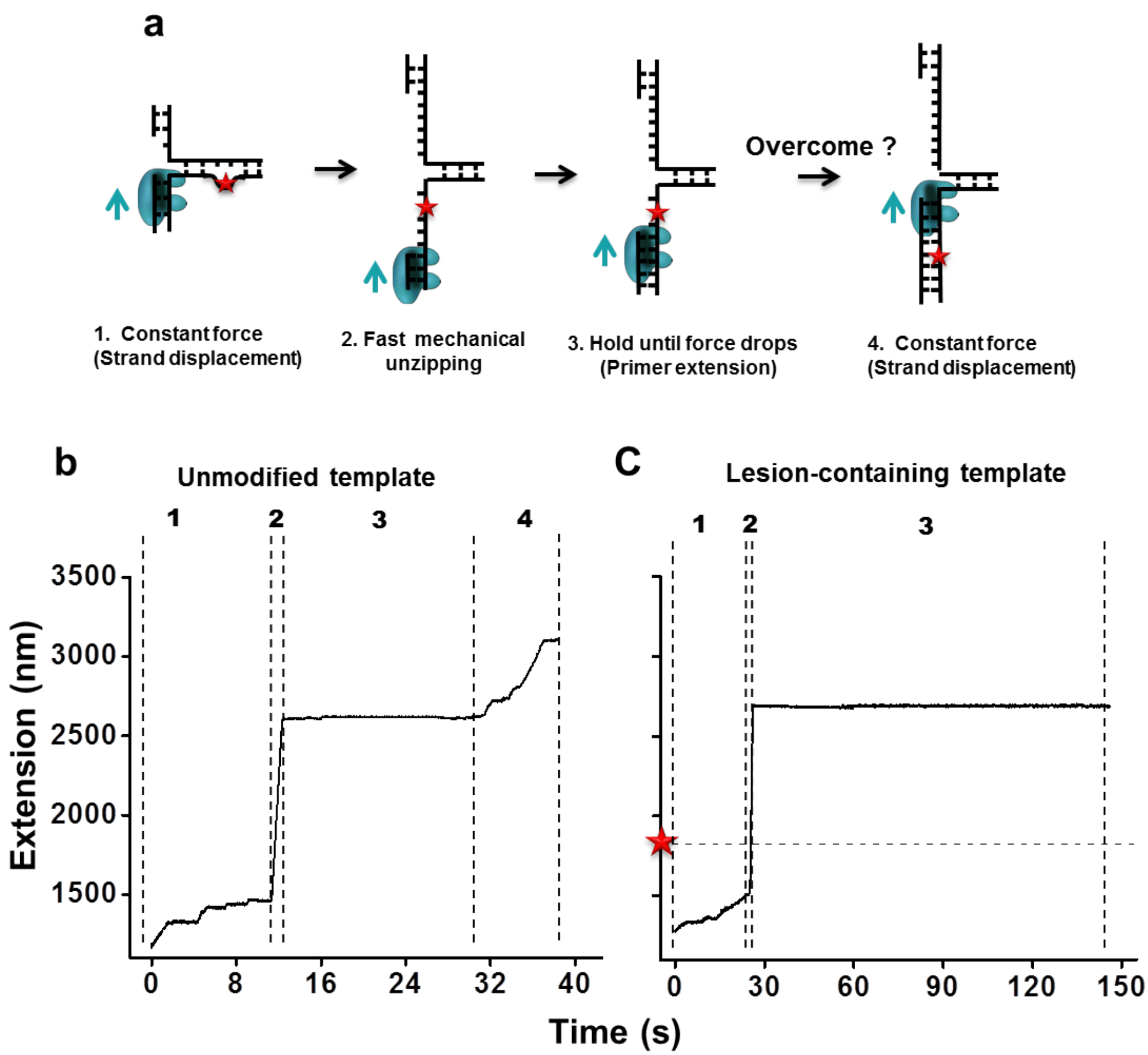

Supplementary Figure 4: Single-molecule studies of primer extension by wt T7 DNAP on unmodified and CPD lesion-containing DNA templates.

a, Schematic representation of the single-molecule configuration of T7 DNAP primer extension on a lesion-containing DNA template. To rule out the possibility that the wt T7 DNAP could not synthesize through the CPD lesion due to the presence of the DNA fork in the strand displacement configuration (Fig. 2a), we conducted a single-molecule primer extension assay on a lesion-containing DNA template. The experimental procedures were: (1) maintain constant force on DNA arms at 12 pN while wt T7 DNAP performs strand displacement synthesis for tens

of seconds; (2) mechanically unzip ~1200 bp of DNA past the lesion (red star) in one second to generate lesion-containing ssDNA; (3) maintain the DNA extension while DNAP is extending the primer in which the fork is no longer a barrier, until force drops indicating T7 DNAP is displaying strand displacement synthesis; (4) maintain constant force at 12 pN while T7 DNAP performs possible strand displacement synthesis again. If wt T7 DNAP is capable of bypassing the lesion, its strand displacement activity will be detected at the fork in step (4).

b, A representative trace of wt T7 DNAP primer extension on an unmodified DNA template in single-molecule studies. Using an unmodified DNA template, we always detected a force dropping within ~17 seconds at the end of step (3) and an extension increasing during step (4). This result indicates wt T7 polymerase is able to perform primer extension to the fork when the DNA arms are held under tension. The primer extension rate is around 70 nt/s under this condition, consistent with previous studies[2].

c, A representative trace of wt T7 DNAP primer extension on a CPD lesion-containing DNA template in single-molecule studies. The horizontal dotted line indicates the lesion position. Using a lesion-containing DNA template, no strand displacement synthesis in step 4 was observed in 2 minutes for all traces, suggesting that wt DNAP was blocked by the lesion during primer extension.

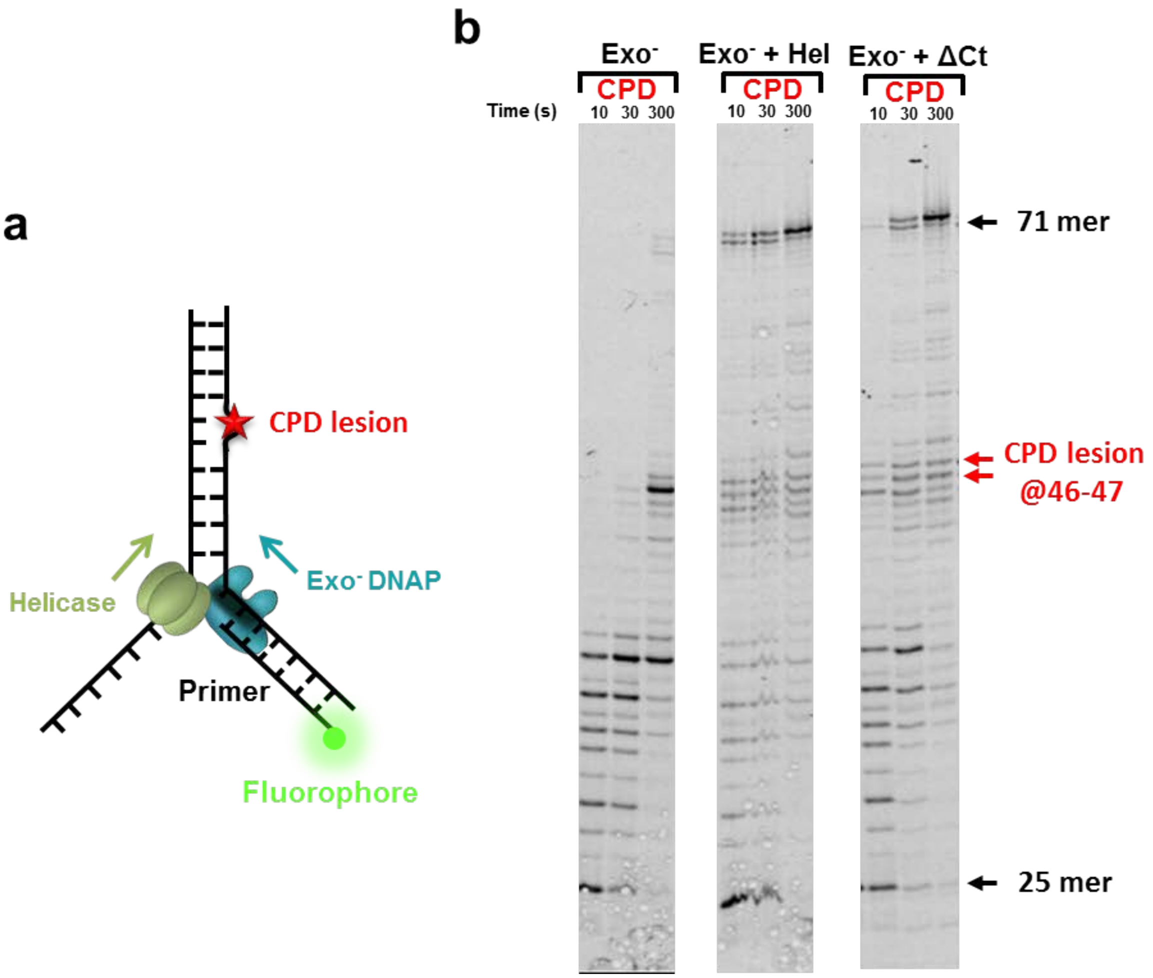

Supplementary Figure 5. Bulk studies of leading-strand synthesis through a DNA template containing a lesion by exo- T7 DNAP in the presence of helicase.

a, Schematic representation of bulk studies of helicase/exo- DNAP coupled leading-strand replication on a CPD lesion-containing template. A 25-mer primer with 5' fluorescein labeled was annealed to the template 71-mer which contains a CPD lesion (red star) at the 46th and 47th nucleotides from the 3' end.

b, Exo- T7 DNAP synthesizing on a CPD lesion-containing template by itself or with T7 helicase/ΔCt mutant. Sequencing gel shows the speed of the leading-strand DNA synthesis.

Reactions were carried out at 18°C using 50 nM DNA, 65 nM T7 exo$^-$ DNAP, and 65 nM T7 helicase hexamer/ ΔCt mutant (see Methods). Exo$^-$ DNAP alone showed slow rate of DNA synthesis, and only 1.8% replicated through the lesion (≥ 48 nt) in 300 seconds. However, up to 53% and 51% of them were able to synthesize through the lesion (≥ 48 nt) in the presence of wt T7 helicase and ΔCt mutant, respectively. These results are expected as exo$^-$ T7 DNAP alone is capable of synthesizing through a CPD lesion in primer extension assays (Supplementary Fig. 2). Once helicase was provided for DNA unwinding in this assay, exo$^-$ T7 DNAP was observed to overcome the lesion.

Supplementary References

1. Inman, J.T. et al. DNA Y structure: a versatile, multidimensional single molecule assay. *Nano Lett* **14**, 6475-80 (2014).
2. Wuite, G.J., Smith, S.B., Young, M., Keller, D. & Bustamante, C. Single-molecule studies of the effect of template tension on T7 DNA polymerase activity. *Nature* **404**, 103-6 (2000).
